# Severe airway stenosis associated with Crohn's disease: Case report

**DOI:** 10.1186/1471-2466-6-7

**Published:** 2006-04-07

**Authors:** Maria Plataki, Eleni Tzortzaki, Irene Lambiri, Elpida Giannikaki, Armin Ernst, Nikolaos M Siafakas

**Affiliations:** 1 Department of Thoracic Medicine, University Hospital of Heraklion, Heraklion, Greece; 2 Department of Pathology, University Hospital of Heraklion, Heraklion, Greece; 3 Department of Medicine, Beth Israel Deaconess Medical Center, Boston, USA

## Abstract

**Background:**

Symptomatic respiratory tract involvement is not common in Crohn's disease. Upper-airway obstruction has been reported before in Crohn's disease and usually responds well to steroid treatment.

**Case presentation:**

We report a case of a 32-year old patient with Crohn's disease who presented with progressively worsening dyspnea on exertion. Magnetic Resonance Imaging of the chest and bronchoscopy revealed severe tracheal stenosis and marked inflammation of tracheal mucosa. Histopathology of the lesion showed acute and chronic inflammation and extended ulceration of bronchial mucosa, without granulomas. Tracheal stenosis was attributed to Crohn's disease after exclusion of other possible causes and oral and inhaled steroids were administered. Despite steroid treatment, tracheal stenosis persisted and only mild symptomatic improvement was noted after 8 months of therapy. The patient subsequently underwent rigid bronchoscopy with successful dilatation and ablation of the stenosed areas and remission of her symptoms.

**Conclusion:**

Respiratory involvement in Crohn's disease might be more common than appreciated. Interventional pulmonology techniques should be considered in cases of tracheal stenosis due to Crohn's disease refractory to steroid treatment.

## Background

Crohn's disease (CD) is a nonspecific chronic inflammatory disease that most commonly affects the distal ileum and colon and occasionally exhibits extra-intestinal manifestations. Symptomatic respiratory tract involvement is not common, pulmonary lesions, however, have been reported in CD, including chronic bronchitis [1], bronchiectasis [1,2], upper-airway obstruction [3-7], bronchiolitis obliterans organizing pneumonia [1], granulomatous lung disease [1,8,9] and interstitial lung disease [10]. This report describes a young patient with CD who developed severe tracheal stenosis.

## Case presentation

A 32-yr old Caucasian female patient presented in September 2004 with progressively worsening dyspnea on exertion. Since 2002, she had suffered from typical CD with arthritis, which had been diagnosed on clinical, endoscopic and histological grounds. She had undergone colonoscopy and biopsy in order to investigate symptoms of persistent diarrhea with abdominal pain, weight loss and fever. Colonoscopy had revealed mucosal ulcerations involving the distal ileum and right colon alternating with areas of normal-appearing mucosa and histological examination had shown transmural inflammation and epithelioid granulomas, typical of CD. In the following years, her gastrointestinal symptoms were well-controlled on sulphasalazine 3000 mg·day^-1^, azathioprine 100 mg·day^-1 ^and folic acid 5 mg·day^-1 ^and she experienced no exacerbations. Over the past year she has suffered from recurrent episodes of acute bronchitis. She has been repeatedly treated with antibiotics and short courses of oral steroids, followed by improvement of symptoms of infection (cough and sputum production). She complained, however, of progressive deterioration of her dyspnea and was referred by her family doctor to the Thoracic Department of the University Hospital of Heraklion for further evaluation and treatment.

**Figure 1:**
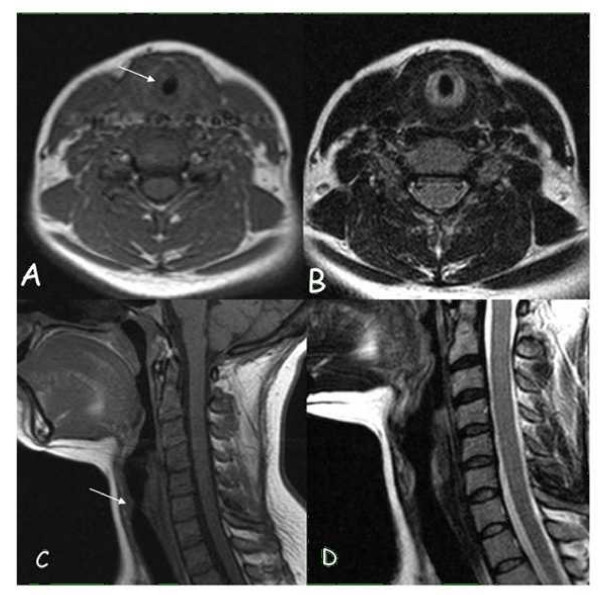
Magnetic Resonance Imaging (MRI). MRI_axial: (A) Axial T1-weighted Spin Echo (SE) MRI shows circumferential thickening of the trachea (white arrow) with intermediate signal intensity of the thickened mucosa/submucosa. (B) T2-weighted Turbo SE MRI at the same level. Note the pathological high signal intensity of the thickened mucosa/submucosa, which corresponds to mucosal inflammation. MRI_sag: (C) Sagittal T1-weighted SE MRI shows irregular narrowing of the airway (white arrow). (D) Sagittal T2-weighted Turbo SE MRI of the subglottic trachea shows thickening of the tracheal lumen with high signal intensity due to inflammation.

On presentation, severe dyspnea on exertion subsiding at rest was noted. On physical examination, the patient was tachypnoeic and had stridor, reduced breath sounds and diffuse wheezing on auscultation. She was a lifelong non-smoker and had no history of occupational or environmental exposure relevant to lung disease. Her temperature was 36.6°C and her arterial oxygen saturation at rest was 97% (room air). Post exertion arterial blood-gas analysis did not reveal hypoxemia (alveolar-arterial oxygen tension gradient at peak exercise was 10 mmHg). The electrocardiogram and chest radiographs were normal. Laboratory investigations showed normal serum values of electrolytes, glucose, protein, liver function tests, urea and creatinine. Other laboratory tests showed a moderately increased erythrocyte sedimentation rate of 31 mm·1 h^-1^, and normal C-reactive protein (< 0.32 mg/dl). Complete blood count revealed mildly elevated white blood cell count (12.3 K·μl^-1^, 90.8% neutrophils). Assessments of anti-nuclear antibodies, anti-DNA antibodies, anti-neutrophil cytoplasmic antibodies (ANCA, both cytoplasmic- and perinuclear-ANCA), and serum angiotensin-converting enzyme (sACE) were normal. Serologic tests for Mycoplasma pneumoniae, Chlamydia pneumoniae, respiratory syncytial virus, adenovirus, and influenza viruses were all negative. Tuberculin skin test was negative. Sputum cultures for mycobacteria and fungi were negative. Pulmonary function tests showed airway obstruction (Table 1) and flow-volume curve intra-extrathoracic airway obstruction. There was no improvement in forced expiratory volume in one second (FEV_1_) following inhalation of β_2_-agonist. Magnetic resonance imaging (MRI) of the trachea revealed a 2-cm region of severe subglottic tracheal stenosis with marked increase in tracheal wall thickness (Figure 1). High resolution computed tomography scan (HRCT) of the thorax did not show any abnormalities.

**Figure 2:**
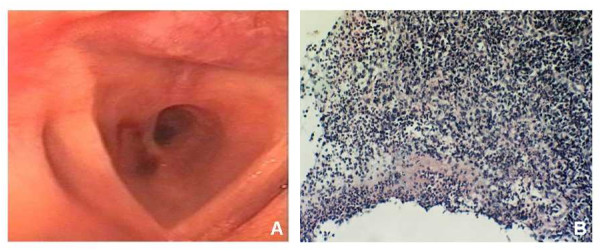
Fiberoptic bronchoscopy and histological examination of tracheal stenosis. (A) Circumferential tracheal stenosis, greater than 70%, 1–2 cm beneath the vocal cords and marked inflammation of tracheal mucosa was noted at fiberoptic bronchoscopy. (B) Histological examination revealed acute and chronic inflammation and extended ulceration of bronchial mucosa, without granulomas.

A fiberoptic bronchoscopy (FOB) was performed, and showed severe circumferential tracheal stenosis, greater than 70%, 1–2 cm beneath the vocal cords and marked inflammation of tracheal mucosa (Figure 2A). The patient did not tolerate penetration of the bronchoscope through the obstruction. Bacteriologic studies of bronchial washings, including cultures for mycobacteria and fungi, were negative. Cytologic studies of bronchial washings showed inflammatory cells and no malignant cells. Histological examination of several biopsies of the stenosed areas revealed acute and chronic inflammation and extended ulceration of bronchial mucosa, without granulomas (Figure 2B).

The patient received inhaled budesonide 800 μg·day^-1 ^and methylprednisolone 120 mg·day^-1 ^iv for 5 days. Then she continued on oral methylprednisolone 24 mg·day^-1 ^and inhaled budesonide 800 μg·day^-1^. During the following months she reported improvement of her shortness of breath, accompanied by gradual improvement of her lung function tests (Table 1). In May 2005 she was referred to the Complex Airway Center of the Beth Israel Deaconess Medical Center for further evaluation.

**Figure 3:**
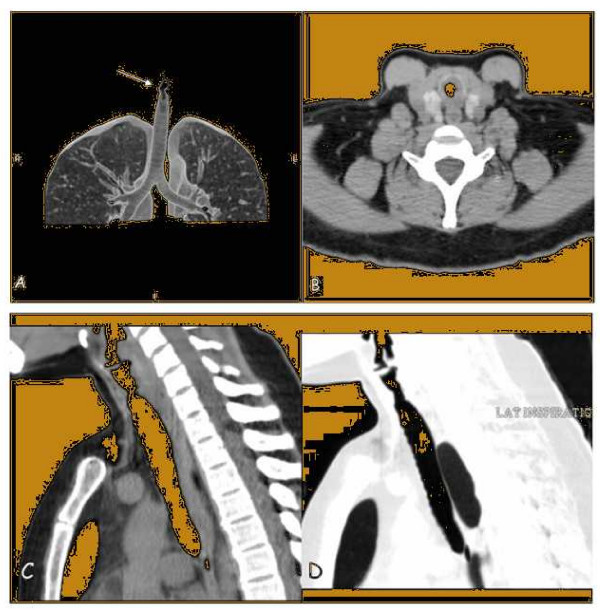
Computed Tomography (CT). (A) CT_ volume rendering technique (VRT): The VRT of the tracheobronchial tree, clearly demonstrates the segment of irregular stenosis of the subglottic trachea (white arrow). (B) CT _axial: CT scan shows marked circumferential narrowing of the subglottic part of the trachea. No calcification can be seen. (C and D) CT_sag_multi planar reconstruction (MPR): On (C) end-expiration and (D) end-inspiration sagittal MPR of CT scan, a dynamic collapse of the trachea can not be documented.

On presentation in the Complex Airway Center, her throat on auscultation revealed mild inspiratory stridor. There was otherwise no wheezing, chest exam was normal, as was the remainder of her examination, and room air oxygenation was 99%. A specialized airway CT was performed, which demonstrated a 2-cm region of circumferential subglottic stenosis with focal narrowing that did not spare the membranous portion of the trachea (Figure 3). The remainder of the trachea appeared unremarkable without evidence of tracheobronchomalacia. There were regions of patchy ground glass opacity in the superior segment of the right middle lobe, with some centrilobular nodular opacities and patchy opacities in the right lower lobe superior segment with air trapping.

At flexible bronchoscopy a high-grade subglottic obstruction was visible at about 7 or 8 mm diameter opening. The obstruction was complex, reaching from the posterior subglottic area close to the vocal cords and posterior arytenoids all the way down to the cricoid ring in a circular fashion. Rigid bronchoscopy confirmed the subglottic stenosis and showed that it extended into the first tracheal ring. The area was biopsied, dilated sequentially to 12 mm and the remaining tissues were cleanly ablated with the help of a microdebrider. The diameter achieved in the end was approximately 14 mm and appeared normal for her size. Five cc of 0.04% mitomycin-C was also applied for four minutes. During the rigid bronchoscopy several additional abnormalities were visualized. The left upper lobe was near-totally occluded by a web-like obstruction, which was also biopsied and dilated. Moreover, the right medial segment of the right lower lobe was near-totally occluded with what appeared to be mucosal and submucosal swelling, which was again extensively biopsied and a lumen was reestablished. The pathology results revealed partially ulcerated respiratory mucosa with squamous metaplasia, lamina propria fibrosis, recent hemorrhages and acute and chronic inflammation in all three locations, but no granulomas.

This treatment led to complete remission of the patient's respiratory symptoms up to the point that this report is written, 10 months after dilatation. She continued treatment with oral methylprednisolone 24 mg·day^-1^, inhaled budesonide 800 μg·day^-1 ^and oral omeprazole 20 mg·day^-1^. The patient will be closely followed with pulmonary function tests and endoscopic approaches, if necessary, and progressive tapering of oral steroid treatment over the following months will be attempted.

## Conclusion

The authors report a case of a young female with CD, who developed tracheal inflammation and stenosis that improved but persisted, despite corticosteroid treatment. Although the lungs are not a common site of involvement, pulmonary lesions have been reported in CD. Latent pulmonary involvement might be more frequent than considered and subclicinal abnormalities, like increased bronchial hyperresponsiveness [11,12], impairement of lung function parameters [13,14], increased lymphocyte numbers in bronchoalveolar lavage and induced sputum [15,16] and increase of lung permeability [17], have been well-described.

In the present case, other disease processes that might cause a similar picture had to be excluded. Tuberculous stenosis is an endobronchial complication of pulmonary tuberculosis. In this patient, mycobacterial infection could be excluded by the absence of caseating granulomas, negative tuberculin skin test, and failure to identify mycobacteria on culture. Sarcoidosis and CD share pathologic and immunologic features [18]. In this case, there was little evidence in favor of sarcoidosis: there was no extrapulmonary sarcoid involvement, no mediastinal lymph node involvement, sACE level was normal and typical sarcoid granulomata in bronchial biopsies were absent. Moreover, the absence of histological findings suggestive of Wegener's granulomatosis, the lack of cytoplasmic-ANCA and no extrapulmonary involvement, made the diagnosis of Wegener's granulomatosis unlikely. Relapsing polychondritis was excluded on the basis of clinical, radiological and histological criteria.

Tracheobronchial involvement is rare in CD, but has been reported before [1-9,19]. Mucosal inflammation of the trachea and bronchi may be observed, with or without upper airway stenosis, in any age group. Histologically, there may be airway infiltration by inflammatory cells and mucosal ulceration, but non-caseating granulomas are not always present [1,8]. The diagnosis of CD usually precedes the presentation of pulmonary manifestations, but previously undiagnosed CD presenting with airway manifestations has also been reported [4]. Symptoms of tracheobronchial involvement usually respond well to appropriate doses of inhaled and/or systemic steroids. In the present case, however, although the patient reported improvement with steroid treatment, she still complained of symptoms compromising her quality of life, making a more invasive approach necessary. Bronchoscopic dilatation led to complete remission of her symptoms. Stent placement was not feasible as the lesion was in the glottic and subglottic space involving the vocal cords. The lesions were not approachable surgically as they were multifocal and still revealed chronic inflammation. Surgery could be indicated at a later point when the inflammation has turned into fibrosis. Aggressive treatment with inhaled and oral steroids is still necessary for this patient to avoid relapse, since inflammation is an ongoing process.

In summary, this is a report of a patient with CD who developed severe airway obstruction, despite the fact that gastrointestinal symptoms were well-controlled on conventional CD treatment. Systemic and inhaled steroid treatment led to some improvement, in agreement with previous reports of the effectiveness of steroid administration in similar cases [1]. In this case however, mucosal inflammation and symptoms persisted, making a more invasive approach necessary. Interventional pulmonary medicine has made great progress during recent years, proving to be a valuable tool for respiratory physicians. To our knowledge this is the first report of a patient with tracheal stenosis from CD successfully treated with rigid bronchoscopy and dilatation. Respiratory involvement in CD might be more common than appreciated and the need exists for early diagnosis and appropriate treatment of airway complications, which might otherwise prove to be life-threatening.

## Abbreviations

anti-neutrophil cytoplasmic antibodies (ANCA), Crohn's disease (CD), fiberoptic bronchoscopy (FOB), forced expiratory volume in one second (FEV_1_), high resolution computed tomography scan (HRCT), magnetic resonance imaging (MRI), serum angiotensin-converting enzyme (sACE)

## Competing interests

The author(s) declare that they have no competing interests.

## Authors' contributions

MP and ET were responsible of patient management and drafted the manuscript. IL carried out flexible bronchoscopy and participated in patient follow-up. EG carried out pathology tests. AE was responsible of all interventional pulmonology procedures. NMS critically revised the manuscript and gave final approval of the version. All authors read and approved the final manuscript.

## Pre-publication history

The pre-publication history for this paper can be accessed here:



## Figures and Tables

**Table 1:** Sequential evaluation of pulmonary function tests.

Parameter	September 2004	October 2004	December 2004	February 2005	July 2005
FVC	3.47 (102)	3.59 (106)	3.56 (106)	3.71 (107.5)	3.88 (112.3)
FEV_1_	1.28 (43.5)	1.6 (54.3)	2.76 (94.5)	3.19 (106.5)	3.39 (113)
FEV_1_/FVC%	36.9	44.5	77.6	86.05	87.42
FEF_25–75 _L/s	1.13 (29.2)	1.41 (36.4)	2.72 (71.1)	3.51 (91.1)	4.00 (103.7)

Data in parentheses are percentage of predicted value. Measurements in September 2004 were taken on admission; those in October 2004 and December 2004 were after 4 weeks and 12 weeks of oral and inhaled steroids, respectively; those in February 2005 were after 20 weeks of therapy; and those in July 2005 after bronchoscopic dilatation and 40 weeks of therapy. FVC: forced vital capacity; FEV_1_: forced expiratory volume in one second; FEF_25–75_: forced mid-expiratory flow.
